# Anisotropic Swelling Behavior of Liquid Crystal Elastomers in Isotropic Solvents

**DOI:** 10.3390/nano15060443

**Published:** 2025-03-14

**Authors:** Limei Zhang, Hong Li, Wenjiang Zheng, Yu Zhao, Weimin Pan, Niankun Zhang, Jing Xu, Xuewei Liu

**Affiliations:** 1 School of Exercise and Health Sciences, Xi’an Physical Education University, Xi’an 710068, China; 230401004@stu.xaipe.edu.cn (Y.Z.); 103022@tea.xaipe.edu.cn (W.P.); 104009@tea.xaipe.edu.cn (N.Z.); 103028@tea.xaipe.edu.cn (J.X.); 2 Engineering Research Center of Innovative Technology of Intelligent Sports Equipment, Universities of Shaanxi Province, Xi’an 710068, China; 3 School of Mechanical Engineering, Xi’an Aeronautical Institute, Xi’an 710077, China; 202309003@xaau.edu.cn; 4 School of Chemical Engineering, Sichuan University of Science and Engineering, Zigong 643000, China; zhengwenjiang@suse.edu.cn; 5 School of Mechanical and Electrical Engineering, Xi’an University of Architecture and Technology, Xi’an 710055, China

**Keywords:** liquid crystal elastomers, isotropic solvents, swelling, anisotropy, phase transition

## Abstract

The chemical response of liquid crystal elastomers (LCEs) offers substantial potential for applications in propulsion systems, micromechanical systems, and active smart surfaces. However, the shape-changing behaviors of LCEs in response to organic (isotropic) solvents remain scarcely explored, with most research focusing on liquid crystal (anisotropic) solvents. Herein, we prepared a series of aligned LCEs with varying crosslink densities using a surface alignment technique combined with an aza-Michael addition reaction, aiming to investigate their swelling behaviors in different isotropic solvents. We found that the rates of shape and volume variation modes, the elastic modulus of the LCEs, and the polarity of the solvent all significantly influence the swelling behavior. Specifically, when LCEs swell in acetone, dimethylformamide (DMF), and ethyl acetate, contraction occurs along the alignment direction. Conversely, extension along the alignment direction is observed when LCEs swell in toluene, anisole, and acrylic acid. Meanwhile, extension in the perpendicular direction is noted when LCEs swell in nearly all solvents. These shape changes can be attributed to the phase transitions of the LCEs. This research not only provides valuable insights into the swelling mechanisms of LCEs but also holds great promise for the development of solvent sensors and gas sensing applications.

## 1. Introduction

Development of soft stimuli responsive materials that can change their properties and performances under ambient conditions is of growing interest in recent years, due to their potential applications in soft robotics, tissue engineering, biosensing, and flexible displays [1,2,3,4]. In nature, shape-changing behaviors are widely observed among various plants and animals. A well-known example is the Venus flytrap, which autonomously closes its leaves in response to mechanical stimuli [5]. Similarly, pinecones exhibit adaptive behavior by opening or closing their scales based on the surrounding humidity. This phenomenon is attributed to the presence of two types of tissues containing cellulose fibers orientated in different directions, leading to anisotropic absorption or desorption of water [6]. Inspired by these natural phenomena, researchers have developed a variety of synthetic polymers, including hydrogels, shape-memory polymers, and LCEs. The responsive shape changes in these materials arise from non-equilibrium matter transport or phase transitions triggered by external stimuli [7,8].

LCEs are a unique class of soft, polymeric, stimulus-responsive actuating materials [9,10]. They are composed of elongated monomers known as mesogens, which self-assemble into liquid crystal structures. These structures are embedded within a lightly crosslinked elastomer network. This synergistic combination endows LCEs with both the long-range orientational order characteristic of liquid crystals and the elastic properties inherent to polymer networks. LCEs exhibit significant and reversible shape changes in response to various external stimuli, including light [11,12,13], heat [14,15,16], electric fields [17,18], and magnetic fields [19]. These shape changes are driven by phase transitions between the nematic and isotropic phases [20]. The nematic phase is defined by a well-ordered arrangement of mesogens, whereas the isotropic phase is characterized by a disordered state. The transition between two phases results in macroscopic deformations in the LCE material.

The chemical responsive behavior of LCEs is also attractive in relation to a variety of potential applications, such as propulsion systems [21], micromechanical systems [22,23,24], and active smart surfaces [25]. However, despite its potential, the shape-changing behaviors of LCEs in response to organic (isotropic) solvents remain scarcely explored, with most studies focusing on liquid crystal (anisotropic) solvents [26,27,28,29,30]. In this study, we explored the swelling behaviors of nematic LCEs in response to different isotropic solvents. A series of LCEs with varying crosslink densities were prepared using a surface alignment technique combined with an aza-Michael addition reaction. The results reveal that the swelling characteristics of LCEs are significantly influenced by the rates of shape and volume variation modes, the elastic modulus of the LCEs, and the polarity of the solvent. When swelling occurs, the macroscopic dimension of the LCEs undergoes a change, which is accompanied by a phase transition. The phase transition was verified through polarizing optical microscope (POM) and wide-angle X-ray diffraction (WAXD) analyses. Meanwhile, the shape-changing behavior was visually recorded using an optical microscope. The study of the anisotropic swelling behavior of LCEs will prompt them to function as smart materials in response to the solution environment and will further accelerate the development of shape-controllable devices, chemo-responsive actuators, and gas sensors.

## 2. Materials and Methods

### 2.1. Materials

Photoresist (S-1813) and photoresist developer (MF-319) were purchased from Shipley Company (Marlborough, MA, USA). Poly (dimethylsiloxane) (PDMS) precursor and curing agent (Sylgard 184) were purchased from Dow Corning (Midland, MI, USA). Epoxy resin (D.E.R. 354) was purchased from Dow Chemical Company (Midland, MI, USA). The liquid crystal monomer 1,4-Bis-[4-(6-acryloyloxyhexyloxy) benzoyloxy]-2-methylbenzene (RM82) was purchased from Wilshire Technologies Inc. (Princeton, TX, USA). Photoinitiator 2,2-dimethoxy-2-phenylacetophenone (DMPA), (tridecafluoro-1,1,2,2-tetrahydrooctyl) trichlorosilane (Silane-8174), n-butylamine, ethylenediamine, toluene, anisole, and ethyl acetate were purchased from Sigma Aldrich (Louis, MO, USA). Acetone and DMF were purchased from Sinopharm Chemical Reagent Co., Ltd. (Shanghai, China). Acrylic acid, isopropyl alcohol, and ethanol were purchased from Guangdong Guanghua Technology Co., Ltd. (Shantou, China). All reagents were used in their received state without further purification.

### 2.2. Fabrication of Liquid Crystal Cells

The liquid crystal cells were fabricated through molecular-scale soft imprint lithography to prepare micro-pattern features. The templates with designed patterns were fabricated from photoresist S-1813 using direct laser writing (Heidelberg DWL 66FS laser lithography system, Heidelberg Instruments, Heidelberg, Germany). Silicon (Si) wafer was pre-cleaned by rinsing with acetone and isopropyl alcohol, followed by drying with an air gun. A thin layer of S1813 with a thickness of approximately 2 μm was spin-coated at 4000 rpm for 40 s onto the clean Si wafer substrate, followed by prebaking at 90 °C for 5 min. The photoresist S1813 layer was then exposed to a direct writing laser (365 nm UV light) with a micro-channel pattern. After laser writing, the sample was developed using photoresist developer (MF-319) to obtain the final S1813 pattern. The wafer with desired patterns was then rinsed twice in deionized (DI) water and finally blow-dried.

A PDMS mold was replicated from the wafer template following the procedure reported earlier [31,32]. Subsequently, the micro-channel patterns were replicated from the PDMS mold onto an epoxy film. Then, a drop of approximately 10 µL of epoxy liquid (D.E.R. 354) was initially placed on a clean glass, followed by placing the patterned PDMS mold on top. The epoxy liquid was allowed to capillary-infiltrate into the PDMS pattern, and then it was exposed to 365 nm UV light at an intensity of 40 mW/cm^2^ for 2 h to fully cure the epoxy. The resulting epoxy replica was carefully peeled off from the PDMS mold.

The as-prepared epoxy patterns were cleaned in an oxygen plasma system (Model 144AX, Jelight Company, Irvine, CA, USA) for 15 min to slightly oxidize the surface. The samples were then transferred to a vacuum chamber for fluoro-silane treatment via a chemical vapor deposition process. A small drop (approximately 5 µL) of fluoro-silane (Silane-8174) was placed at the center of the vacuum chamber, surrounded by epoxy samples at a similar distance from the center droplet. The chamber was then vacuum-pumped for 30 min, and the final fluoro-silane-coated (F-coated) samples were kept in a dry place for future use.

The F-coated epoxy substrates were used as both the top and the bottom surfaces for constructing liquid crystal cells with an area of 1.5 × 1.5 cm^2^. The top and bottom channels of the LC cells were assembled so that they were parallel to each other. The thickness of the LC cells was controlled by using double-faced adhesive tape (70 μm thick, 3M Company, Paul, MN, USA) as the spacer.

### 2.3. Synthesis and Preparation of the Aligned LCEs

The aligned LCEs were synthesized through aza-Michael addition-based step-growth polymerization. Liquid crystalline monomer (RM82, *x* mol), chain extender (n-butylamine, *y* mol), and crosslinker (ethylenediamine, (*x* − *y*)/2 mol) were mixed together in a sealed glass vial. A series of aligned main-chain nematic LCEs with various crosslink densities were prepared. By controlling the molar ratio of crosslinkers to liquid crystal monomer ($w_{EDA}$) at 20 mol%, 30 mol%, and 40 mol%, the corresponding molar ratio of chain extenders to liquid crystal monomer was 60 mol%, 40 mol%, and 20 mol%, respectively. The mixture was then heated to fully melt and vigorously vortexed for uniform mixing. Subsequently, the mixture was capillary-infiltrated into the liquid crystal cell on a hot stage at 80 °C within 10 min. Then, the cell was transferred to an oven at 80 °C for 24 h, which allowed the aza-Michael addition reaction between diacrylate and amine to undergo chain extension and crosslinking. When the cell had been slowly cooled down to room temperature under ambient conditions, it was opened using a razor blade, and the epoxy alignment layers were carefully peeled off to obtain a freestanding LCE sheet for later use.

### 2.4. Gel Fraction

The LCEs were immersed in toluene for 48 h to remove any un-crosslinked components. Subsequently, the samples were dried in a vacuum oven at room temperature for 12 h. The mass of the samples was measured both before and after toluene extraction, and the gel fraction values (%) were then calculated by

$$gel\;fraction\;(\%)\;=\;\frac{m_{e}}{m_{0}}\times 100\%,$$

where $m_{0\;}$ is the initial mass of the LCEs before extraction and $m_{e}$ is the final dried mass of the LCEs after extraction. The experiments were performed three to five times, and the results were averaged out.

### 2.5. Mechanical Properties

The mechanical properties of the LCEs were characterized using a dynamic mechanical analyzer (DMA Q800, TA instruments, New Castle, PA, USA) equipped with a tension film clamp. During the tensile measurements, the samples were first equilibrated at 25 °C under a preload of 0.01 N. The force was then increased at a rate of 0.2 N/min to evaluate the mechanical behavior of the materials.

### 2.6. Optical Microscopy

A POM (BX61, Olympus, Tokyo, Japan) equipped with a heating stage and CellSens software (Version 4.2) was used to determine the phase transitions, alignment, and deformation of the LCEs. The nematic–isotropic transition temperatures (T_NI_) of the liquid crystal mixtures were confirmed by cooling the samples from the isotopic phase. Qualitative analyses of the order parameter of LCEs in both swollen and un-swollen states were conducted using a POM fitted with crossed polarizers. The dimensional changes in the LCE films in response to solvents were monitored via an optical microscope. The LEC film was cut into a rectangular strip. The dimensions of the planar LCE samples in their original aligned state, when immersed in solvents, and after drying, were visualized under the microscope. The dimensions of the films, which remained constant at each state, were quantified from the images by using the CellSens software. The relative change in length was determined by

$$deformation\;quantity\;(\%)\;=\;\frac{L}{L_{0}}\times 100\%$$

where *L*_0_ is the initial length of the sample, *L* is the length of the sample at each respective steady state. The measurements were conducted three to five times, and the results were averaged to ensure reliability.

### 2.7. Wide-Angle X-Ray Diffraction

Two-dimensional (2D) WAXD patterns were acquired utilizing a Rigaku X-ray imaging system coupled with an 18 kW rotating anode X-ray generator. An exposure time of at least 20 min is necessary to achieve patterns of adequate quality. The background scattering was subsequently subtracted from the sample scans to enhance the clarity and accuracy of the diffraction data.

## 3. Results and Discussion

### 3.1. Fabrication of the Liquid Crystal Cells

**Figure 1:**
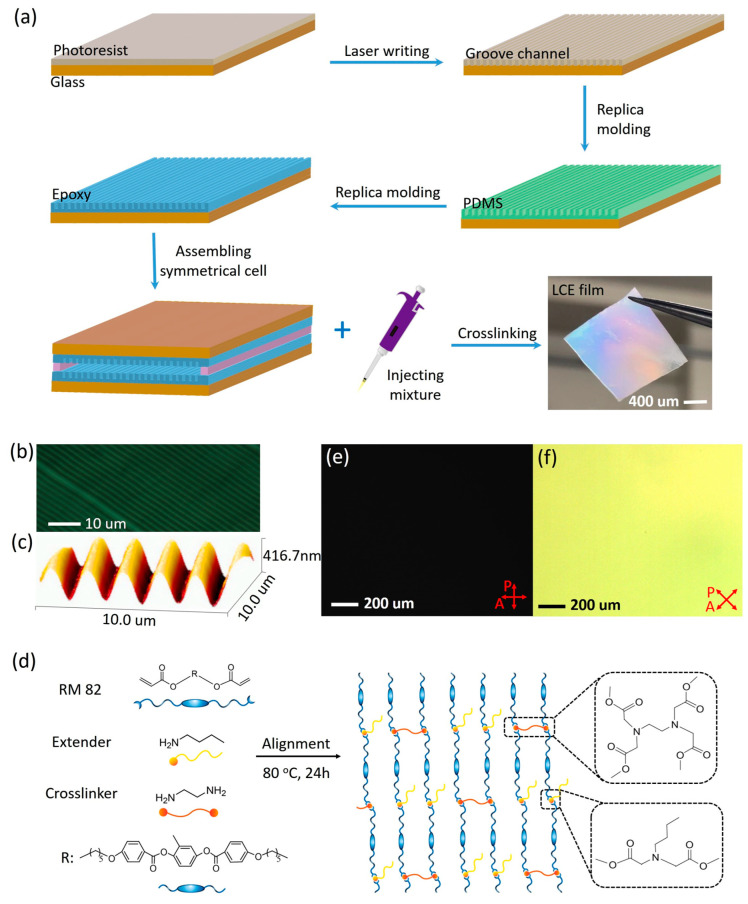
Fabrication of aligned LCE films by polymerization of liquid crystal monomers within cells. (**a**) Molecular-scale soft imprint lithography is used to prepare liquid crystal cells with uniform micro-channel patterns, thereby aligning LCEs. (**b**) Optical microscope images and (**c**) AFM images of the micro-channels on the surface of the liquid crystal cell. (**d**) Chemical structure and synthetic process of the LCEs. POM images of the LCE film (40 mol% $w_{EDA}$ sample) measured between crossed polarizers, (**e**) with the direction of the aligned LCEs parallel to the analyzer (0°) and (**f**) with the direction of the aligned LCEs at a 45° angle to the analyzer.

We employed a surface alignment technique to achieve the alignment of the mesogenic units within liquid crystal cells. The cells were fabricated using soft imprint lithography to create micro-patterned features (Figure 1a). First, a silicon wafer substrate was coated with photoresist, which was then exposed to a direct writing laser to form micro-channel patterns. The alignment of the mesogenic units within these micro-channels is determined by the anchoring strength [33]. When the anchoring strength is maximized, the mesogens achieve a stable orientation with their directors aligned parallel to the micro-channel direction [34]. The small pattern feature size, with a width of 1 µm, provides a high surface anchoring strength (Figure 1b). Second, a PDMS mold was replicated from the wafer template following a previously reported procedure [31,32]. Then, the micro-channel patterns were transferred from the PDMS mold to an epoxy film. To enhance alignment, a chemical vapor deposition process was used to create fluoro-silane coated (F-coated) samples. The interactions between the mesogens and the substrate surface also play a crucial role in inducing alignment. Finally, the F-coated epoxy substrates were used to construct liquid crystal cells. Atomic force microscopy (AFM) images revealed that the channel-patterned molds had a width of 1 µm, spacing of 1 µm, and a depth of approximately 400 nm (Figure 1c). The two sides of the mold were placed with a defined spacing, into which the molten mixture was injected. The liquid crystal mesogens rapidly aligned to match the micro-channel patterns of the cell. Further details are provided in Section 2.

### 3.2. Preparation of the Aligned LCEs

The aligned main-chain LCEs were synthesized via an aza-Michael addition reaction within the liquid crystal cells [35,36]. The aza-Michael addition reaction is an efficient synthetic route for preparing LCEs, primarily due to its suitable kinetics and high conversion rates. This reaction mechanism is analogous to the classic Michael addition, where a nitrogen-containing nucleophile attacks the β-carbon of an α, β-unsaturated carbonyl compound, leading to the formation of a new carbon–nitrogen bond. Its relatively slow polymerization and crosslink kinetics are particularly advantageous for surface alignment techniques used in preparing aligned LCEs. These kinetics provide sufficient time for the mesogens to fully align within the low-viscosity reaction mixture, ensuring optimal molecular orientation [37]. We have developed an aza-Michael addition reaction combined with a surface alignment technique to fabricate aligned nematic LCEs using amine and diacrylate components. This method leverages slow polymerization and crosslink kinetics to align the mesogens in liquid crystal cells. The alignment is then maintained by extending the heat treatment time to complete the aza-Michael addition reaction, ultimately yielding well-aligned main-chain nematic LCEs. In brief, n-butylamine (chain extender), ethylenediamine (crosslinker, EDA), and the liquid crystal mesogen 1,4-bis[4-(6-acryloyloxyhexyloxy)benzoyloxy]-2-methylbenzene (RM82) were homogeneously mixed and filled into the cell. The liquid crystal mesogen rapidly achieved full orientation. Subsequently, the cell was placed in an oven at 80 °C for 24 h, with the temperature set within the nematic phase of the mixture (Figure 1d). During this period, the aza-Michael addition reaction between the diacrylate and amine groups resulted in step-growth polymerization, yielding aligned nematic LCEs. The optical images of the LCEs are shown in Figure 1a.

A series of aligned main-chain nematic LCEs with various crosslink densities were prepared. The molar ratio of crosslinkers to liquid crystal monomer, denoted as $w_{EDA}$, was set to 20 mol%, 30 mol%, and 40 mol%. Correspondingly, the molar ratio of the chain extender to liquid crystal monomer was adjusted to 60 mol%, 40 mol%, and 20 mol%, respectively. The nematic-to-isotropic phase transition temperatures (T_NI_) of the liquid crystal mixtures with $w_{EDA}$ values of 20 mol%, 30 mol%, and 40 mol% were 84 °C, 87 °C, and 92 °C, respectively. These values, determined using an optical microscope, gradually increased with higher $w_{EDA}$ content, attributed to the increasing crosslink density [38]. The gel fractions for the 20 mol%, 30 mol%, and 40 mol% $w_{EDA}$ samples were 93.85 ± 2.57%, 95.76 ± 2.3%, and 97.23 ± 1.66%, respectively, confirming sufficient crosslink in the LCEs. The macroscopic alignment of the planarly aligned LCEs was verified by POM, as shown in Figure 1e,f. The aligned LCEs (40 mol% $w_{EDA}$ sample) were rotated and observed under an optical microscope equipped with crossed polarizers. As the samples were rotated, alternating bright and dark states were observed. The maximum brightness was achieved when the sample was orientated at a 45° angle to the polarizer, while the darkest state was observed at 0°. The spatial uniformity of the images in these two states indicates the good, homogeneous in-plane alignment of the LCEs.

### 3.3. Mechanical Properties of the LCEs

**Figure 2:**
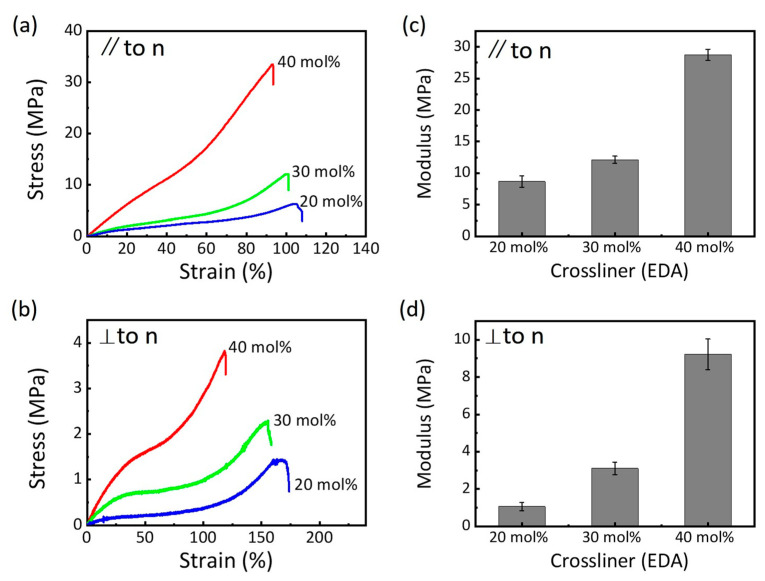
Mechanical properties of the LCEs. Stress–strain curves of LCEs with various $w_{EDA}$, with loading applied (**a**) parallel and (**b**) perpendicular to the director orientation. Modulus of LCEs with various $w_{EDA}$, with loading applied (**c**) parallel and (**d**) perpendicular to director orientation. The nematic director is denoted as “n”.

The mechanical properties of the LCEs were examined through uniaxial elongation tests at room temperature, with loading applied in two different directions relative to the director orientation (Figure 2). The dynamic mechanical analyzer was employed to obtain the stress–strain curves of the LCEs, leveraging its high sampling frequency to capture the detailed mechanical behavior. The mechanical properties of LCEs are significantly influenced by the molar ratio of the crosslinker ($w_{EDA}$). As $w_{EDA}$ increases from 20 mol% to 40 mol%, the fracture stress and modulus of the LCEs rise, while the fracture strain decreases. This trend is consistent regardless of whether the loading is applied along or perpendicular to the director orientation. The increase in mechanical properties can be attributed to the higher polymer chain density and crosslink density associated with increased $w_{EDA}$. These factors enhance the load-bearing capacity of the LCEs but reduce their deformation capacity. When loaded along the director orientation, the LCEs exhibit stiff and relatively brittle behavior, characterized by a higher modulus and lower fracture strain (Figure 2a,c). In contrast, when loaded perpendicular to the director, the LCEs display compliant characteristics, with a lower modulus and higher fracture strain (Figure 2b,d). This anisotropic mechanical response is closely related to the molecular alignment of the mesogens within the LCEs. The director orientation plays a crucial role in determining the deformation and stress distribution under uniaxial tensile loading and further influences the material’s overall mechanical behavior. Figure 2b illustrates the characteristic soft-elastic plateaus observed in the stress–strain curves of LCEs when stretched perpendicular to the director orientation. These plateaus, which appear at nearly 50% strain, are indicative of the material’s unique mechanical adaptability. This phenomenon is closely associated with the reorientation of the director along the loading direction, allowing the LCEs to undergo large deformations before failure. This soft-elastic behavior is a typical characteristic of LCEs and is attributed to the reorientation of mesogens within the material, which can accommodate significant strain without substantial increases in stress. The extent of the soft-elastic region in LCEs is influenced by the crosslink density, as demonstrated by the $w_{EDA}$ content. For instance, the 20 mol% $w_{EDA}$ samples (with lower modulus, Figure 2d) allow for the greater reorientation of the director and exhibit a more pronounced soft-elastic plateau, spanning from 10% to 90% strain. In contrast, the 40 mol% $w_{EDA}$ samples (with higher modulus, Figure 2d) display a narrower soft-elastic region, ranging from 40% to 60% strain. This difference is attributed to the increased network rigidity in the higher $w_{EDA}$ samples, which restricts the reorientation of mesogens and limits the material’s ability to undergo large deformations. This relationship underscores the importance of tuning the crosslink density to optimize the mechanical properties of LCEs for specific applications. Additionally, previous studies have shown that other factors, such as temperature and strain rate, can also influence the soft-elastic behavior of LCEs [39]. These findings confirm the critical roles of crosslink density and director orientation in tailoring the mechanical properties of LCEs. This understanding provides a foundation for designing LCE-based materials with optimized performance for specific applications, such as actuators and soft robotics.

### 3.4. Anisotropic Swelling Behavior of the LCEs

**Figure 3:**
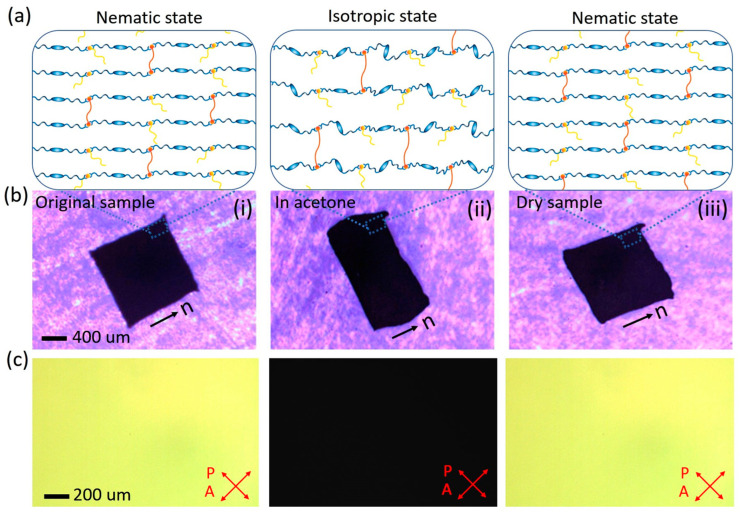
Swelling behavior of the LCE films in acetone. (**a**) Schematic illustration of the network structure and (**b**) optical images of the dimensions and (**c**) POM images of the LCEs in different states: (**i**) original aligned state, (**ii**) immersed in acetone, and (**iii**) after drying. The LCE films were orientated at a 45° angle with respect to the cross polarizers. A 20 mol% $w_{EDA}$ sample was used. The “n” represents the nematic director.

LCEs exhibit remarkable shape-changing capabilities, able to rapidly and reversibly alter their form in response to stimuli from the solvent environment. To investigate these shape-changing behaviors, an optical microscope was utilized to track the changes in the planar dimensions of the LCEs, focusing on axes parallel and perpendicular to the director. The change in thickness, in response to solvent stimuli, was assumed to be proportional to the change along the axis perpendicular to the director. The shape-changing behaviors of the LCEs in response to acetone are illustrated in Figure 3. Upon immersion in acetone, the LCEs experienced a reduction in length along the director axis, while simultaneously expanding along the axis perpendicular to the director (Figure 3b). This anisotropy demonstrates that the shape changes are directionally dependent. After the acetone evaporated, the LCEs reverted to their original dimensions. Indicating the reversible property of the swelling behavior. To elucidate the differences in the orientational order of LCEs across various states, we employed an optical microscope equipped with cross polarizers. This technique is particularly effective in visualizing the molecular alignment within LCEs by analyzing the intensity of transmitted light. When the LCE samples were orientated at a 45° angle relative to the polarizers, the brightness of the transmitted light provided a clear indication of the underlying molecular order. Specifically, both the originally aligned sample and the sample after drying exhibited bright states, which are characteristic of the nematic phase (Figure 3c). This brightness arises from the ordered arrangement of mesogens within the LCEs, allowing light to pass through the polarizers without significant attenuation. In stark contrast, the sample immersed in acetone appeared dark, signifying a transition to the isotropic phase. This phenomenon can be attributed to the solvent’s interaction with the LCE molecules. Acetone, as a good solvent for LCEs, effectively disrupts the π-π interactions between the aromatic rings of the LCE molecules. This disruption compromises the molecular orderliness, causing the LCEs to temporarily transition from the nematic phase to the isotropic phase, where the mesogens are randomly orientated. As a result, the transmitted light is significantly attenuated, leading to the observed dark appearance under cross polarized microscopy. This observation underscores the critical role of solvent interactions in modulating the molecular order of LCEs and confirms the reversible nature of the phase transition between the nematic and isotropic phases. The order–disorder transition not only alters the material’s microscopic structure but also results in significant macroscopic shape changes. Upon exposure to acetone, the LCEs transition from a highly ordered nematic state (unswollen) to a slightly ordered isotropic or amorphous state (swollen) and revert to the nematic state again upon drying (deswelling) (Figure 3a). This demonstrates the reversible and anisotropic swelling behavior of LCEs in response to solvent stimuli.

**Figure 4:**
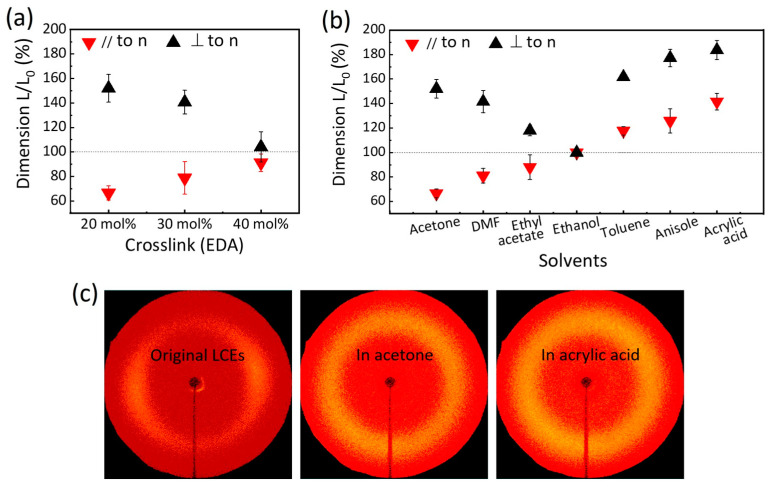
Anisotropic swelling behavior of LCE films in various solvents. (**a**) Deformation of LCEs with different Young’s modulus upon swelling in acetone. (**b**) Deformation of LCEs upon swelling in various solvents. (**c**) The 2D-WAXD patterns of the original aligned LCEs, LCEs swollen in acetone, and LCEs swollen in acrylic acid. The 20 mol% $w_{EDA}$ samples were used.

The network elasticity of LCEs has an effect on their swelling behavior. As the crosslink density or Young’s modulus of the LCEs increases, the degree of swelling gradually decreases, which is specifically manifested by a significant reduction in dimensional changes along all axial directions. For example, the 20 mol% $w_{EDA}$ samples, which possess a lower Young’s modulus, undergo pronounced shape changes when swelling in acetone. Specifically, these samples experience a decrease in length of around 66% along the director axis, while expanding by approximately 152% in the direction perpendicular to the director (Figure 4a). In contrast, the 40 mol% $w_{EDA}$ samples, which have a higher Young’s modulus, exhibit more restrained changes. Their axis along the director contracts to 91% of its original length, and the axis perpendicular to the director expands to 104% of its original length. This result is accordance with previous studies on other polymers [40,41,42]. Based on these findings, we can infer that if the Young’s modulus of the LCEs were to increase further, swelling behavior might no longer occur in acetone.

To systematically investigate the swelling behavior of LCEs in response to different solvents, a range of isotropic solvents were employed, including acetone, DMF, ethyl acetate, ethanol, toluene, anisole, and acrylic acid (Figure 4b). The swelling of LCEs in these solvents exhibits unusual kinetics, which can be attributed to the presence of shape and volume variation modes with markedly different rates [43,44]. We observed that LCEs consistently expand in the direction perpendicular to the director when swelling in the vast majority of solvents. This behavior may be due to the slower rate of shape variation compared to volume variation in the perpendicular direction. In contrast, when LCEs are immersed in acetone, DMF, and ethyl acetate, contraction is observed along the director axis. This suggests that the rate of shape variation exceeds that of volume variation in this direction, leading to contraction rather than expansion. When LCEs are immersed in toluene, anisole, and acrylic acid, dimensions extend along both axes parallel and perpendicular to the director. This indicates that the rate of shape variation is slower than that of volume variation in both directions. Furthermore, the anisotropic mechanical properties of LCEs, characterized by different moduli along axes parallel and perpendicular to the director, result in varying degrees of swelling under a given organic solvent. Additionally, the swelling behavior is strongly influenced by the polarity of the solvent [45]. Notably, swelling in LCEs is minimal in the polar solvent ethanol, emphasizing the significant role of solvent polarity in dictating the swelling behavior of LCEs.

The orientated state of the material is thermodynamically non-equilibrium, and it will spontaneously lose its alignment upon swelling to minimize the system’s energy. To analyze the order parameter of the LCEs, WAXD measurements were performed using a 2D detector, with X-rays irradiated normally to the director of the films. Figure 4c presents representative 2D-WAXD patterns for three distinct states of LCEs, including the original aligned LCEs, LCEs swollen in acetone, and LCEs swollen in acrylic acid. These patterns provide critical insights into the molecular order and alignment of the LCEs at the microscopic level, which directly correlates with their macroscopic shape. In the as-prepared, original aligned LCEs, an intense diffraction arc is observed at wide angles on the equator. This sharp and well-defined arc is indicative of a highly ordered nematic phase, where the mesogens are aligned in a parallel fashion. This molecular alignment is crucial to the anisotropic mechanical properties of the LCEs. Upon swelling in different solvents, significant changes in the 2D-WAXD patterns are observed. For LCEs swollen in acetone or acrylic acid, the once intense equatorial halos become diffuse. This diffusion of the diffraction pattern is attributed to a phase transition from the nematic state to the isotropic state. In the isotropic state, the mesogens lose their ordered alignment and become randomly orientated, leading to a loss of anisotropy. This change in molecular order is consistent with the macroscopic shape changes observed in the LCEs, where the aligned structures transition to a more amorphous and isotropic morphology. The correlation between the molecular-level phase transition and the macroscopic shape response demonstrates the versatility of LCEs in actuating applications. These findings demonstrate that the anisotropic shape response of LCEs can be controlled in actuating applications by selecting different solvents or combinations of solvents.

## 4. Conclusions

In summary, we have systematically investigated the anisotropic swelling behaviors of LCEs in various isotropic solvents. Our study reveals that the swelling behavior of nematic LCEs is highly dependent on the specific solvent used, with distinct shape-changing behaviors observed across different solvents. This behavior can be attributed to several key factors, including the rates of shape and volume variation, the elastic modulus of the LCEs, and the polarity of the solvent. These factors collectively influence the swelling dynamics and the resulting macroscopic shape changes. When LCEs swell in nearly all isotropic solvents, consistent expansion is observed along the axis perpendicular to the director orientation. In contrast, the behavior along the director axis is more complex. Contraction occurs when LCEs swell in solvents such as acetone, DMF, and ethyl acetate, while expansion is seen in solvents like toluene, anisole, and acrylic acid. These macroscopic shape changes are accompanied by phase transitions, as the LCEs transition from a highly ordered nematic state (unswollen) to a slightly ordered isotropic state (swollen) and revert to the nematic state upon drying (deswelling). By understanding the interplay between solvent and LCEs, we can tailor the swelling response to achieve desired mechanical outcomes. This opens up new possibilities for the development of smart materials that can be triggered by specific organic solvents or mixed solvent systems, with potential applications in soft actuators, sensors, and adaptive materials. To further enhance our understanding of these behaviors, we recommend incorporating modeling indications in future work. This will help elucidate the underlying mechanisms and provide a more detailed framework for predicting and optimizing the swelling behavior of LCEs in various solvents.

## Data Availability

The data presented in this study are available upon request from the corresponding author.
